# Predictive Ability of Laboratory Indices for Liver Fibrosis in Patients with Chronic Hepatitis C after the Eradication of Hepatitis C Virus

**DOI:** 10.1371/journal.pone.0133515

**Published:** 2015-07-27

**Authors:** Yoshihiko Tachi, Takanori Hirai, Hidenori Toyoda, Toshifumi Tada, Kazuhiko Hayashi, Takashi Honda, Masatoshi Ishigami, Hidemi Goto, Takashi Kumada

**Affiliations:** 1 Department of Gastroenterology, Komaki City Hospital, Komaki, Japan; 2 Department of Gastroenterology, Ogaki Municipal Hospital, Ogaki, Japan; 3 Department of Gastroenterology and Hepatology, Nagoya University Graduate School of Medicine, Nagoya, Japan; Kaohsiung Medical University Hospital, Kaohsiung Medical University, TAIWAN

## Abstract

Liver fibrosis remains an important risk factor for hepatocarcinogenesis in patients with chronic hepatitis C even after the eradication of hepatitis C virus (HCV). However, it is difficult to estimate liver fibrosis based on liver biopsy after the eradication of HCV. We investigated the ability of laboratory indices to predict liver fibrosis in patients with sustained virologic response (SVR) to antiviral therapy. Three laboratory liver fibrosis indices (aspartate aminotransferase-platelet ratio index (APRI), FIB-4 index, and Forns index) were calculated based on data at the time of initial pretreatment liver biopsy and at second liver biopsy performed approximately 5 years after SVR in 115 patients who underwent serial liver biopsies. The indices at the time of initial biopsy were compared to histological degree of liver fibrosis in initial biopsy, and laboratory indices at the time of second liver biopsy were compared to the degree of fibrosis in second biopsy. In both comparisons, there were significant increases in all 3 indices with the increase of liver fibrosis grade as assessed in liver biopsy specimens. All 3 indices at the time of second biopsy were able to predict moderate to advanced (METAVIR score F2-4) and advanced (F3-4) fibrosis on liver biopsy, with the area under the receiver-operating characteristics curve >0.8 and the accuracy >70%. All 3 laboratory indices of fibrosis accurately reflected liver fibrosis in patients with SVR for 5 years despite the normalization of serum liver transaminase activity and the lack of liver inflammation.

## Introduction

Chronic hepatitis C virus (HCV) infection is a major cause of cirrhosis and hepatocellular carcinoma (HCC) [1–3]. Chronic infection with HCV induces the progression of liver fibrosis, which results in the development of cirrhosis and HCC. The eradication of HCV with antiviral therapy, defined as a sustained virologic response (SVR), will prevent the progression of chronic hepatitis and associated complications [4]. Several studies have reported that achievement of SVR results in the resolution of liver fibrosis [5–7] and a decreased incidence of HCC [8–12]. However, HCC sometimes develops in patients who achieve SVR [13–17], indicating the necessity of continuous surveillance for HCC even after HCV eradication.

Several previous studies have reported that the degree of liver fibrosis is closely associated with the risk of HCC development in chronic hepatitis C patients [10]. Since liver fibrosis remains even after the eradication of HCV, albeit with gradual resolution after SVR, accurate and serial estimation of liver fibrosis is desirable even after HCV eradication. However, performing repeated liver biopsies to evaluate liver fibrosis after SVR is difficult and impractical.

Recently, several laboratory indices of liver fibrosis have been reported [18–27]. The accuracy of these indices in predicting liver fibrosis has been studied in patients with persistent HCV infection. However, whether these indices can identify mild and severe liver fibrosis in patients after the eradication of HCV, in whom serum transaminase activity usually normalizes and liver fibrosis resolves slowly, has not been clarified.

In the present study, we evaluated the accuracy of 3 laboratory liver fibrosis indices, i.e., aspartate aminotransferase-platelet ratio index (APRI), FIB-4 index, and Forns index, as markers of liver fibrosis in patients who achieved SVR and underwent liver biopsies 5 years after HCV eradication.

## Patients and Methods

### Study Patients

**Fig 1:**
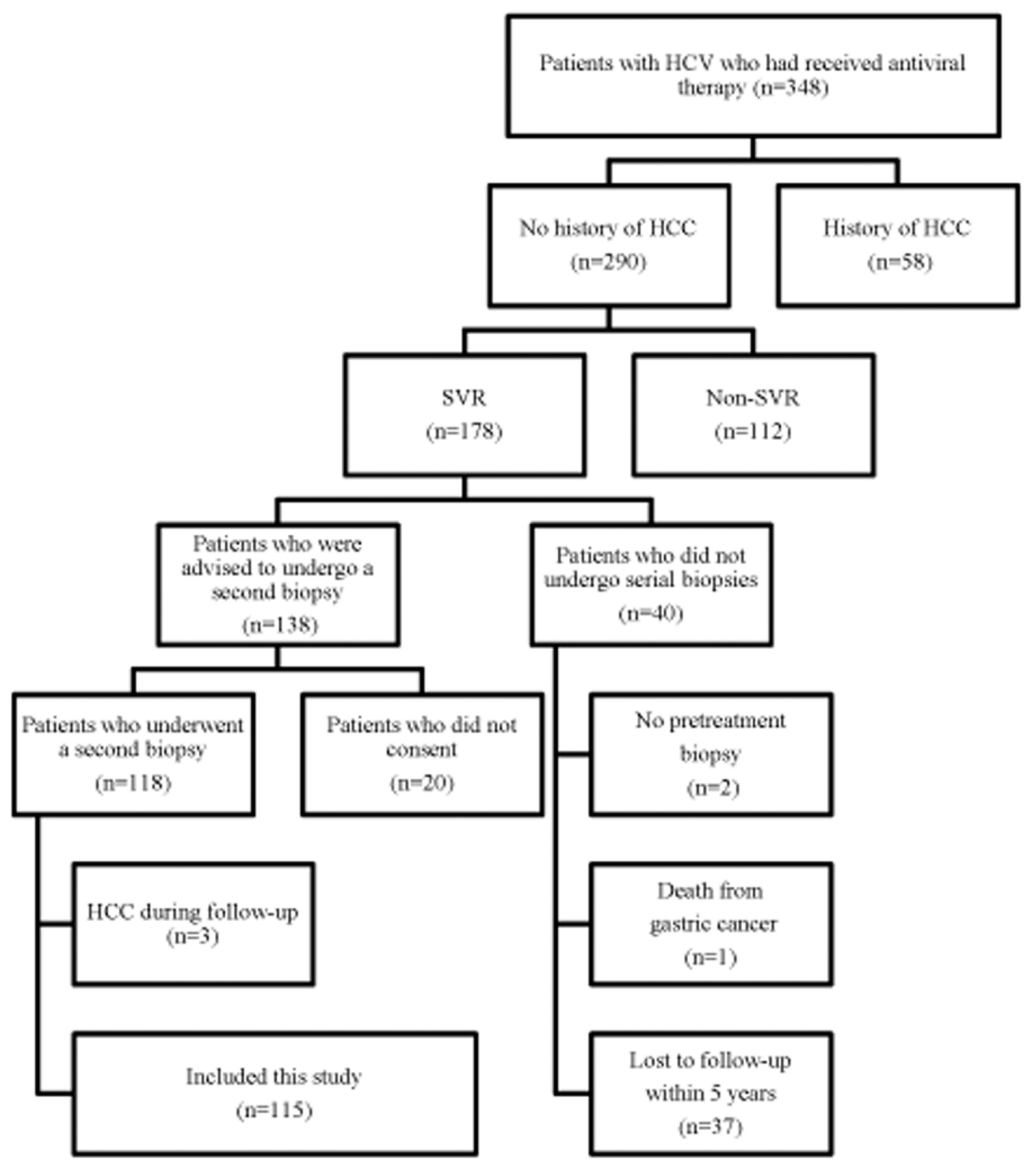
Study flow diagram.

A total of 348 consecutive patients with chronic HCV infection received interferon (IFN)-based antiviral therapy (IFN monotherapy, IFN plus ribavirin combination therapy, or peginterferon (PEG-IFN) plus ribavirin combination therapy) between 1992 and 2008 at Komaki City Hospital. Patients were excluded if they had antibodies against human immunodeficiency virus or hepatitis B virus surface antigen, excessive active alcohol consumption (daily intake > 40 g of ethanol) or drug abuse, or other forms of liver disease (e.g., autoimmune hepatitis, alcoholic liver disease, or hemochromatosis). We excluded 56 patients from the study due to a previous history of HCC. There were 178 patients who achieved SVR, defined as undetectable serum HCV RNA 24 weeks after the completion of antiviral therapy with a real-time PCR assay (COBAS TaqMan HCV test; Roche Molecular Systems: Pleasanton, CA, USA; lower limit of detection, 1.2 log_10_ IU/mL). Of the patients who achieved SVR, 40 patients did not undergo serial biopsies, due to the lack of a pretreatment biopsy (n = 2), death from gastric cancer after SVR (n = 1), or loss to follow-up either by transferring to another hospital or dropping out of the study within 5 years after SVR (n = 37). The remaining 138 patients were scheduled for a protocol-driven second biopsy that was planned approximately 5 years after SVR to investigate changes in liver fibrosis associated with the eradication of HCV, but 20 patients did not provide consent for the second biopsy. Thus, 118 patients underwent the protocol-driven second biopsy, but 3 patients were excluded from the study due to the development of HCC during the follow-up period. Ultimately, 115 patients who achieved SVR were analyzed in the present study (Fig 1).

The entire protocol was approved by the institutional review board of Komaki City Hospital and was carried out in compliance with the Declaration of Helsinki. Written informed consent for liver biopsy and utilization of clinical data was obtained from all patients at the time of the initial and second biopsies.

### Calculation of Laboratory Liver Fibrosis Indices

Blood samples were collected after overnight fasting on the same day as the initial liver biopsy (pretreatment), on the same day as the determination of SVR at 24 weeks after completion of therapy (SVR24), and on the same day as the second biopsy (more than 5 years after therapy). The following values were obtained through serum sample analysis: aspartate aminotransferase (AST), alanine aminotransferase (ALT), gamma-glutamyl transpeptidase (GGTP), platelet count, and cholesterol.

The APRI [18] was calculated as

(AST [IU/L] / upper limit of normal AST [IU/L]) × 100 / platelet count [10^9^/L].

The FIB-4 index [19, 20] was calculated as

AST [IU/L] × age [years]/ platelet count [10^9^/L] × ALT [IU/L]^1/2^.

The Forns index [21] was calculated as

7.811–3.131*ln(platelet count [10^9^/L]) + 0.781*ln(GGT [IU/L]) + 3.467.ln(age)– 0.014*(cholesterol [mg/dL]).

### Histologic Evaluation

The initial biopsy was performed within 2 weeks of the commencement of antiviral therapy, and the second biopsy was performed 5.9 ± 1.8 years after the initial biopsy. Ultrasound-guided, percutaneous needle liver biopsy was performed with a 16-G disposable needle (Bard MAGNAM; C.R. Bard, Murray Hill, New Jersey, USA) in all patients. The median liver biopsy length was 28 mm. The specimens were stained with Hematoxylin-Eosin, Azan, and Gitter stains. A single pathologist, who was blinded to the clinical data, evaluated all the liver biopsy samples. Fibrosis staging scores were assigned according to the METAVIR criteria [28]. Fibrosis was staged on a scale of 0 to 4: F0 (no fibrosis), F1 (portal fibrosis without septa), F2 (few septa), F3 (numerous septa without cirrhosis), or F4 (cirrhosis).

### Statistical Analysis

Quantitative values are reported as means ± SD. Differences in means were analyzed using Student’s *t* test. Changes in the distribution of the liver fibrosis grade between the initial and second liver biopsies were analyzed with the Cochran-Armitage test. Changes in the fibrosis indices with the increase in the liver fibrosis grade were analyzed with Jonckheere-Terpstra test. The predictive performance of the 3 indices was assessed using receiver-operating characteristics (ROC) analysis; the area under the ROC curve (AUROC) was calculated. The point at which the Youden index (sensitivity + specificity– 1) was maximized was used as the cut-off value for each index. Sensitivity, specificity, positive predictive value (PPV), negative predictive value (NPV), and accuracy were calculated for identifying morerate/advanced liver fibrosis (METAVIR F2–4) and advanced liver fibrosis (METAVIR F3–4). Data analysis was performed using JMP statistical software, version 10 (Windows version; SAS Institute, Cary, NC, USA). All *P* values were derived from 2-tailed tests, with *p* <0.05 accepted as statistically significant.

## Results

### Patients Characteristics and Changes in Laboratory Data, Liver Fibrosis Indices, and Liver Histology between Pretreatment Initial Biopsy and Second Biopsy After SVR

Table 1 shows the characteristics of the study patients and laboratory data at the time of the pretreatment initial biopsy and the second liver biopsy 5 years after the achievement of SVR. Patients consisted of 70 (60.9%) males and 45 (39.1%) females, with a mean age of 58.7 ± 9.7 years at initial biopsy. Serum AST, ALT, and GGTP levels were significantly lower at the time of the second biopsy than at the time of the initial biopsy (*p*<0.0001 for all comparisons). Platelet count and serum total cholesterol levels at the time of the second biopsy was significantly higher than values during the initial biopsy (*p*<0.0001 for both comparisons).

**Table 1:** Characteristics of the study patients and changes in laboratory data associated with SVR (n = 115). SVR, sustained virologic response; HCV, hepatitis C virus; APRI, aspartate aminotransferase-platelet ratio index.

Characteristic	At initial pretreatment biopsy^4^	At second biopsy after SVR^5^
Age (years)	58.7 ± 9.7	64.4 ± 9.3
Sex (female/ male)	45 (39.1) / 70 (60.9)	—
HCV genotype (1b/ 2a or 2b)^1^	73 (66.4) / 37 (33.6)	—
*IL28B* polymorphism (TT / GG or TG)^2^	87 (89.7) / 10 (10.3)	—
Alanine aminotransferase (IU/L)	73.8 ± 50.7	18.8 ± 11.6
Aspartate aminotransferase (IU/L)	59.0 ± 41.4	23.1 ± 8.4
Gamma-glutamyl transpeptidase (IU/L)	53.4 ± 60.1	29.0 ± 25.9
Platelet count (x 10^3^/μL)	153 ± 55	175 ± 52
Serum cholesterol (mg/dL)	172.6 ± 28.5	200.8 ± 37.1
APRI	1.43 ± 1.28	0.45 ± 0.24
FIB-4 index	3.27 ± 2.41	2.36 ± 1.33
Forns index	6.79 ± 1.82	5.82 ± 1.60
Liver fibrosis (F0 / F1 / F2 / F3 / F4)^3^	3 (2.6) / 66 (57.4) / 31 (27.0) / 12 (10.4) / 3 (2.6)	29 (25.2) / 61 (53.0) / 9 (7.8) / 11 (9.6) / 5 (4.4)

^1^HCV genotype was not assessed in 5 patients.

^2^ *IL28B* polymorphism was not assessed in 18 patients.

^3^Based on the METAVIR scoring system.

^4^At the time of the initial liver biopsy, before antiviral therapy.

^5^At the time of the second liver biopsy, more than 5 years after the determination of SVR. Among 12 patients with F3 fibrosis at the pretreatment initial biopsy, fibrosis grade decreased to F1 in one patient and F2 in two patients at the second biopsy. Among 3 patients with F4 fibrosis at the pretreatment initial biopsy, fibrosis grade decreased to F3 in one patient at the second biopsy. Among 11 patients with F3 fibrosis at the second biopsy, fibrosis grade was F2 at the pretreatment initial biopsy and increased in two patients. Among 5 patients with F4 fibrosis at the second biopsy, fibrosis grade was F2 in two patients and F3 in one patient at the pretreatment initial biopsy.

Regarding liver fibrosis indices, all 3 indices (APRI, FIB-4 index, Forns index) were significantly lower at the time of the second biopsy compared to the time of the initial biopsy (*p*<0.0001 for all comparisons). There was a shift toward lower histological fibrosis grades in the second biopsy compared to the initial biopsy (*p*<0.0001). As for patients with advanced fibrosis (F3-4), fibrosis grade decreased from F3 to F1 in one patient, from F3 to F2 in two patients, and from F4 to F3 in one patient at the second biopsy from the pretreatment initial biopsy. In contrast, fibrosis grade increased from F2 to F3 in two patients, F2 to F4 in two patients, and F3 to F4 in one patient at the second biopsy from the pretreatment initial biopsy. Among these 5 patients in whom liver fibrosis progressed, one patient had diabetes and 3 patients had obesity (body mass index >25.0). No patients had habitual alcohol intake. Hepatic steatosis was observed at second biopsy in 4 patients.

### Association of Laboratory Liver Fibrosis Indices Calculated at the Time of the Pretreatment Initial Liver Biopsy with Histological Liver Fibrosis Grade and Predictive Performances for Moderate to Advanced (F2-4) and Advanced (F3-4) Fibrosis

**Fig 2:**
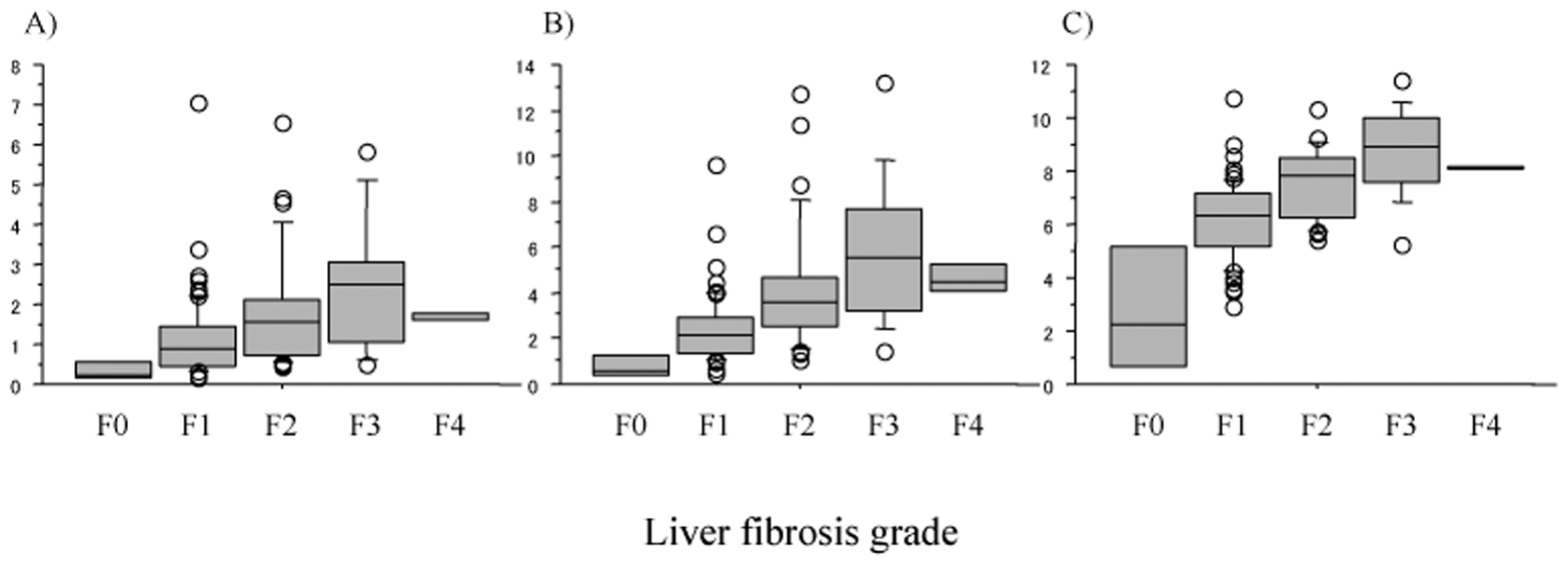
Laboratory liver fibrosis indices calculated on data at the time of the pretreatment initial liver biopsy based on the histological fibrosis in the initial biopsy. (A) The aspartate aminotransferase-platelet ratio index (APRI), (B) FIB-4 index, and (C) Forns index. There are significant increases of all 3 indices with the increase in histological liver fibrosis (all, *p*< 0.0001).

**Fig 3:**
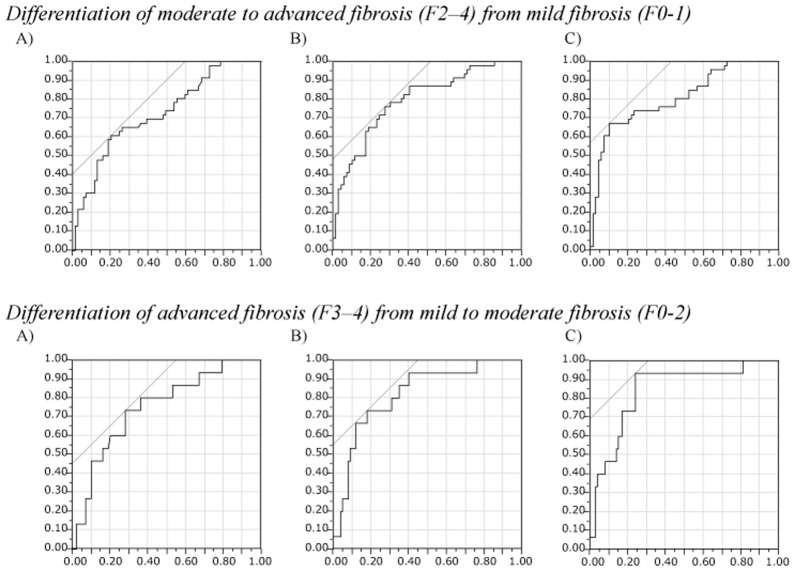
Receiver-operating characteristics (ROC) analysis of laboratory indices of liver fibrosis calculated with laboratory data at the time of the pretreatment initial liver biopsy for identifying moderate to advanced fibrosis (F2–4, upper panel) and advanced fibrosis (F3-4, lower panel) of the liver in the initial biopsy. (A) The aspartate aminotransferase-platelet ratio index (APRI), (B) FIB-4 index, and (C) Forns index. Vertical axis, Sensitivity; horizontal axis, 1 –specificity.

There were significant increases in all 3 laboratory liver fibrosis indices calculated at the time of the pretreatment initial liver biopsy along with the increase in the histological liver fibrosis grade of the initial liver biopsy (all, *p*<0.0001; Fig 2). The ROC analysis for each fibrosis index in identifying moderate/advanced (F2-4) and advanced (F3-4) liver fibrosis (Fig 3) showed that all 3 indices had good predictive ability for both moderate/advanced and advanced liver fibrosis with AUROCs above 0.7 and with the accuracy above 70% (Tables 2 and 3). Although there were minor differences in the sensitivity, specificity, PPV, NPV, and accuracy between the 3 indices, their ability to predict moderate/advanced and advanced fibrosis was comparable.

**Table 2:** Parameters associated with the use of 3 laboratory indices of liver fibrosis calculated on data at the time of the initial liver biopsy prior to interferon-based therapy for differentiating moderate to advanced histological liver fibrosis (METAVIR F2–4) from mild liver fibrosis (METAVIR F0–1) of the liver on initial biopsy (n = 115). APRI, aspartate aminotransferase-platelet ratio index; AUROC, Area under the ROC curve; PPV, positive predictive value; NPV, negative predictive value.

Fibrosis marker	AUROC	Cut-off	Youden index	Sensitivity (%)	Specificity (%)	PPV (%)	NPV (%)	Accuracy
APRI	0.7262	1.54	0.4058	58.7	79.7	65.9	74.3	71.3
FIB-4 index	0.7940	2.84	0.4855	73.9	72.5	64.2	80.6	73.0
Forns index	0.8088	7.56	0.5725	65.2	89.9	81.1	79.5	80.0

**Table 3:** Parameters associated with the use of 3 laboratory indices of liver fibrosis calculated on data at the time of the initial liver biopsy prior to interferon-based therapy for differentiating advanced histological liver fibrosis (METAVIR F3–4) from mild to moderate liver fibrosis (METAVIR F0–2) of the liver on initial biopsy (n = 115). APRI, aspartate aminotransferase-platelet ratio index; AUROC, Area under the ROC curve; PPV, positive predictive value; NPV, negative predictive value.

Fibrosis marker	AUROC	Cut-off	Youden index	Sensitivity (%)	Specificity (%)	PPV (%)	NPV (%)	Accuracy
APRI	0.7503	1.58	0.4533	73.3	72.0	28.2	94.7	72.2
FIB-4 index	0.8200	3.97	0.5533	66.7	83.0	37.0	94.3	80.9
Forns index	0.8400	7.56	0.6933	86.7	76.0	35.1	97.4	77.4

### Association of Laboratory Liver Fibrosis Indices Calculated at the Second Liver Biopsy 5 Years after SVR with Histological Liver Fibrosis Grade and Predictive Performances for Moderate to Advanced (F2-4) and Advanced (F3-4) Fibrosis

**Fig 4:**
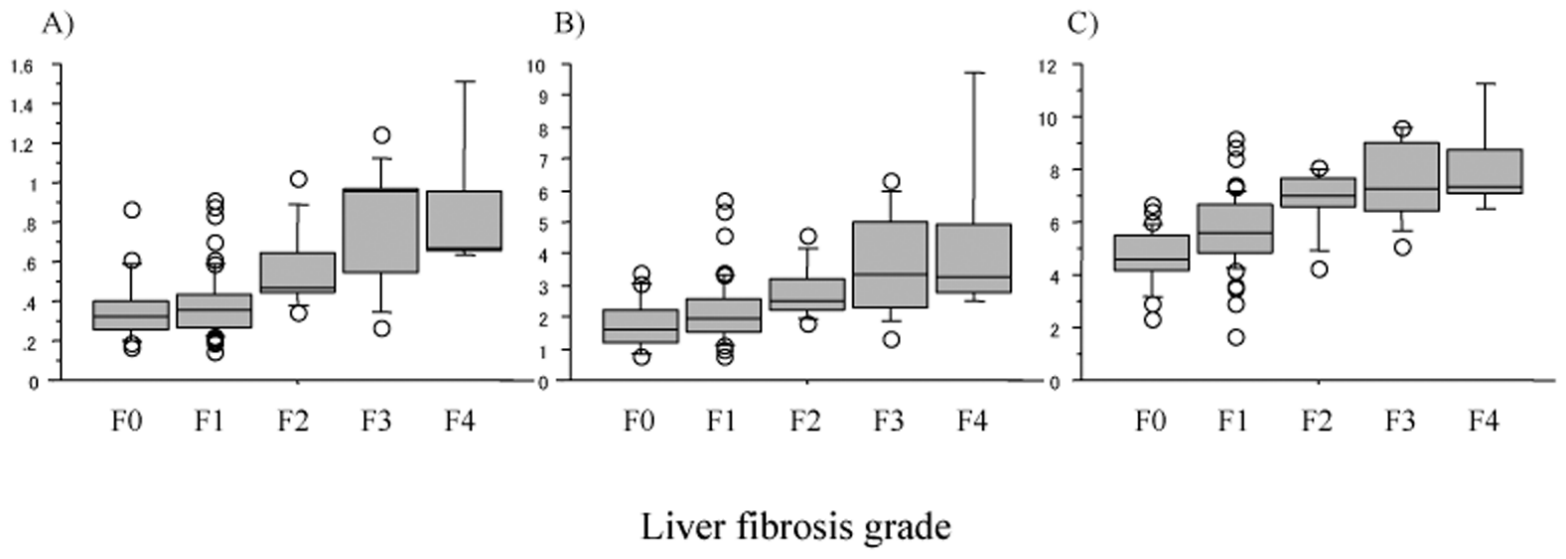
Laboratory liver fibrosis indices calculated on data at the time of post-SVR second liver biopsy (5 years after SVR) based on the histological fibrosis in the post-SVR second biopsy. (A) The aspartate aminotransferase-platelet ratio index (APRI), (B) FIB-4 index, and (C) Forns index. There are significant increases of all 3 indices with the increase in histological liver fibrosis (all, *p*< 0.0001). SVR, sustained virologic response

**Fig 5:**
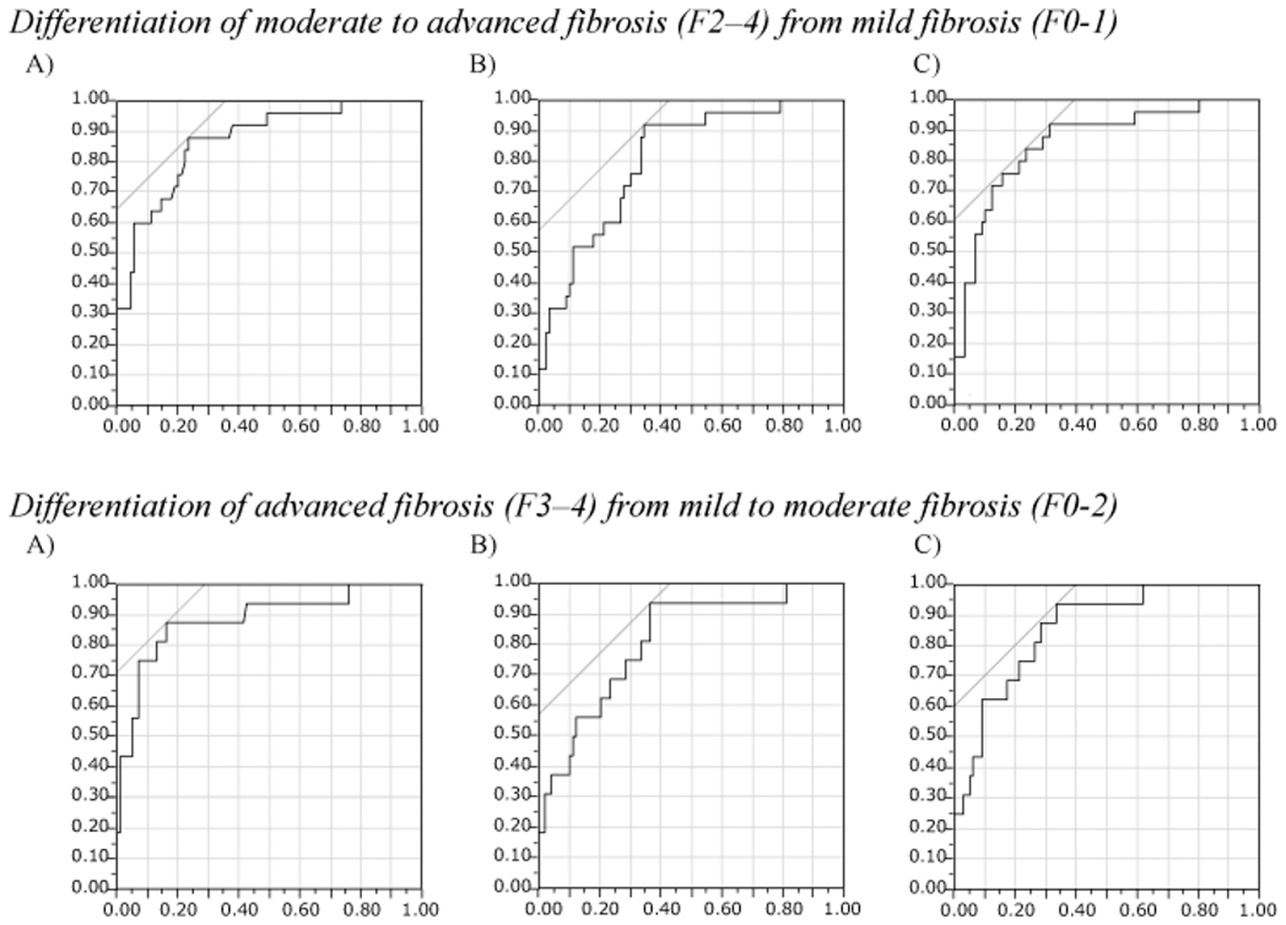
Receiver-operating characteristics (ROC) analysis of laboratory indices of liver fibrosis calculated with laboratory data at post-SVR second liver biopsy for identifying moderate to advanced fibrosis (F2–4, upper panel) and advanced fibrosis (F3-4, lower panel) of the liver in the second liver biopsy. (A) The aspartate aminotransferase-platelet ratio index (APRI), (B) FIB-4 index, and (C) Forns index. Vertical axis, Sensitivity; horizontal axis, 1 –specificity. SVR, sustained virologic response

There were significant increases in all 3 laboratory liver fibrosis indices calculated on the data at the second liver biopsy 5 years after SVR, i.e., eradication of HCV, along with the increase in the histological liver fibrosis grade of the post-SVR second liver biopsy (all, *p*<0.0001; Fig 4). The ROC analysis for each fibrosis index in identifying moderate/advanced (F2-4) and advanced (F3-4) liver fibrosis (Fig 5) showed that all 3 indices had good predictive ability for both moderate/advanced and advanced liver fibrosis of the pretreatment liver with AUROCs above 0.8 and with the accuracy above 70%, except for FIB-4 index for the prediction of advanced fibrosis (67.8%) (Tables 4 and 5). Although there were minor differences in the sensitivity, specificity, PPV, NPV, and accuracy between the 3 indices, their ability to predict moderate/advanced and advanced fibrosis was comparable.

**Table 4:** Parameters associated with the use of 3 laboratory indices of liver fibrosis calculated on data at the time of the second liver biopsy (more than 5 years after SVR) for differentiating moderate to advanced histological liver fibrosis (METAVIR F2–4) from mild liver fibrosis (METAVIR F0–1) of the liver on the post-SVR second biopsy (n = 115). SVR, sustained virologic response; APRI, aspartate aminotransferase-platelet ratio index; AUROC, Area under the ROC curve; PPV, positive predictive value; NPV, negative predictive value.

Fibrosis markers	AUROC	Cut-off	Youden index	Sensitivity (%)	Specificity (%)	PPV (%)	NPV (%)	Accuracy
APRI	0.8696	0.43	0.6467	88.0	76.7	51.2	95.8	79.1
FIB-4 index	0.8067	2.22	0.5756	92.0	65.6	42.6	96.7	71.3
Forns index	0.8604	5.97	0.6089	92.0	68.9	45.1	96.9	73.9

**Table 5:** Parameters associated with the use of 3 laboratory indices of liver fibrosis calculated on data at the time of the second liver biopsy (more than 5 years after SVR) for differentiating advanced histological liver fibrosis (METAVIR F3–4) from mild to moderate liver fibrosis (METAVIR F0–2) of the liver on the post-SVR second biopsy (n = 115). SVR, sustained virologic response; APRI, aspartate aminotransferase-platelet ratio index; AUROC, Area under the ROC curve; PPV, positive predictive value; NPV, negative predictive value.

Fibrosis markers	AUROC	Cut-off	Youden index	Sensitivity (%)	Specificity (%)	PPV (%)	NPV (%)	Accuracy
APRI	0.8861	0.54	0.7134	81.3	83.8	44.8	96.5	83.5
FIB-4 index	0.8125	2.29	0.5739	93.8	63.6	29.4	98.4	67.8
Forns index	0.8567	6.07	0.6042	93.8	66.7	31.3	98.5	70.4

## Discussion

The emergence of new direct-acting antiviral drugs against HCV will dramatically increase the number of patients who achieve SVR [29–32]. Consequently, there will be an increase in the number of patients who develop HCC after SVR in the near future. Several previous studies have reported residual liver fibrosis as an important risk factor of the development of HCC after the eradication of HCV [13–16]. In addition, our previous study revealed that continuous progression of liver fibrosis was observed even after the eradication of HCV in some patients, and that the likelihood of developing HCC was higher in these patients [33]. Therefore, continued monitoring of liver fibrosis status after the achievement of SVR will be preferable in the management of this patient subpopulation.

Liver biopsy is the gold standard for assessing liver fibrosis [34]. However, it is associated with rare but lethal complications [35], and it is impractical to perform serial liver biopsies after the achievement of SVR. Several surrogate markers for liver fibrosis have been investigated in patients with chronic hepatitis C. Although these markers were confirmed to reflect liver fibrosis in patients with persistent HCV infection, their performances in predicting liver fibrosis in patients who achieved SVR remains unclear. Whereas fibrosis is progressive in patients with persistent HCV infection, fibrosis is usually resolving in patients who have achieved SVR [5–7]. In addition, serum AST and ALT activity is usually normal in patients after achieving SVR. Given these conditions, do these laboratory indices of liver fibrosis retain the ability to predict liver fibrosis?

In the present study, we estimated the accuracy of 3 laboratory fibrosis indices, APRI, FIB-4 index, and Forns index, before and after antiviral therapy in patients who ultimately achieved SVR. Our analysis of the associations between histological liver fibrosis and laboratory indices at the time of the initial pretreatment liver biopsy, corresponding to conditions during persistent HCV infection, confirmed the ability of these 3 indices to predict liver fibrosis as previously reported. For all 3 indices, the AUROC for identifying moderate/advanced liver fibrosis (F2–4) was greater than 0.70 and greater than 0.75 for identifying advanced liver fibrosis (F3–4).

All 3 indices calculated based on laboratory data from the time of the second liver biopsy were also strongly associated with histological findings of liver fibrosis 5 years after the eradication of HCV. The AUROCs for identifying mild liver fibrosis were more than 0.80, and more than 0.75 for severe liver fibrosis, which indicates that these 3 indices can predict moderate/advanced or advanced liver fibrosis, and can be used to estimate liver fibrosis in the long-term after the eradication of HCV, when liver inflammation is usually absent with normal transaminase activity and liver fibrosis is regressing. A recent study by Degasperi et al. [36] reported low predictive values of these fibrosis indices with 0.5 to 0.6 of AUROC in the analysis of 20 patients who achieved SVR. In contrast, our study with larger number of patients showed the ability of these indices to predict both moderate/advanced and advanced liver fibrosis 5 years after the achievement of SVR.

Interestingly, all 3 evaluated laboratory fibrosis indices have comparable ability for predicting liver fibrosis; no index was clearly more or less useful. Although the cut-off values for APRI were markedly lower for laboratory data from the time of the second liver biopsy after SVR due to the normalization of serum transaminase activity, they retained the ability to identify mild and severe fibrosis. This was also observed for the FIB-4 and Forns indices. The formula for the FIB-4 index includes patient age. Although the age of the patients had increased at the second liver biopsy 5 years after SVR, the cut-off levels for identifying moderate/advanced and advanced fibrosis both decreased. However, based on the results of the present study, Forns index may be the best when considering the application of these indices for the estimation of liver fibrosis in both patients with persistent HCV infection (i.e., pretreatment) and patients after the eradication of HCV, because Forns index showed more than 0.8 AUROC for all analyses.

The retention of the ability of these laboratory indices of liver fibrosis to predict histological findings of liver fibrosis suggests that the regression of liver fibrosis can be evaluated without invasive liver biopsies after the eradication of HCV. In addition, estimation of liver fibrosis as surveillance for HCC after SVR may provide information on the risk of HCC development in individual patients within this subpopulation. Therefore, these laboratory indices of liver fibrosis will be useful not only during persistent HCV infection but also after HCV eradication.

However, there is important weakness of these indices of laboratory liver fibrosis. The cut-off values of these indices, especially APRI, to predict moderate to advanced fibrosis or advanced fibrosis were largely different between pretreatment initial biopsy and post-treatment second biopsy. This is owing to the rapid normalization of serum AST, ALT, and GGTP levels with the eradication of HCV. Therefore, it would be difficult to evaluate serial changes liver fibrosis before and after the eradication of HCV directly using these laboratory indices, although changes in the grade of liver fibrosis can be compared before and after the eradication of HCV after the prediction of fibrosis grade using laboratory liver fibrosis indices. Further studies will be necessary for the stability of the cut-off values of these laboratory indices after SVR for the prediction of liver fibrosis based on the duration after the eradication of HCV, and for the ability of these indices to predict serial changes of liver fibrosis after SVR directly.

There are several limitations in this study. The number of study patients was not very large. This was due to the difficulty in obtaining informed consent for invasive liver biopsies among patients who have achieved SVR. In addition, percentage of patients with cirrhosis was low. This was because IFN-based antiviral therapy is not covered by the Japanese National Medical Insurance System for patients who had cirrhosis at the start of the antiviral therapy. The confidence intervals of cut-offs of these laboratory indices to predict histological moderate/advanced and advanced fibrosis after SVR were not evaluated due to the lack of validation study. The validation study would be necessary to confirm the stability for these cut-offs, although it will not be feasible to obtain the dataset for validation. In addition, other reported laboratory liver fibrosis indices, including FibroIndex [22], Fibrometer [23], FibroTest [24], and Hepascore [25], were not evaluated due to the lack of laboratory data needed to calculate these indices. Evaluation of these markers in patients with SVR is necessary for determining the best laboratory markers for estimating the degree of liver fibrosis in patients after HCV eradication in the future. Finally, it should be verified whether these laboratory indices can predict the risk of developing HCC after SVR. Although several previous studies reported the grade of liver fibrosis as one of risk factors for HCC development after SVR in patients with HCV [13–16,37], the usefulness of these laboratory indices of liver fibrosis measured after SVR in the assessment of the risk of HCC development after SVR is to be clarified.

## Conclusion

The laboratory fibrosis indices APRI, FIB-4 index, and Forns index can predict histological moderate/advanced and advanced fibrosis in the liver even after HCV has been eradicated for 5 years, in addition to the livers with persistent HCV infection. These indices will remain useful for estimating liver fibrosis after HCV eradication and may also be useful for assessing the risk of developing HCC after SVR.

## Supporting Information

S1 Table Data at pretreatment biopsy: histological liver fibrosis grade (based on METAVIR criteria), age (years), and laboratory data (AST [IU/L], ALT [IU/L], GGT [IU/L], cholesterol [mg/dL], and platelet count [10^9^/L]) at pretreatment initial liver biopsy. (XLSX)

S2 Table Data at second biopsy after SVR: histological liver fibrosis grade (based on METAVIR criteria), age (years), and laboratory data (AST [IU/L], ALT [IU/L], GGT [IU/L], cholesterol [mg/dL], and platelet count [10^9^/L]) at post-treatment second liver biopsy at 5 years after SVR. (XLSX)
